# Perioperative considerations for patients with multiple myeloma

**DOI:** 10.1097/EA9.0000000000000124

**Published:** 2026-07-08

**Authors:** Tracy Kallenbach

**Affiliations:** From the University of Cape Town, Department of Anaesthesia and Perioperative Medicine, New Somerset Hospital, South Africa

## Abstract

Multiple myeloma is an incurable cancer characterised by clonal plasma-cell proliferation, arising predominantly in the bone marrow but producing widespread systemic complications. Additionally, this cancer usually presents in elderly patients who are often frail and suffering from multiple comorbidities. The global prevalence of multiple myeloma is steadily increasing, with therapeutic advances contributing to improved survival. There are several complications of multiple myeloma that the anaesthetist needs to be aware of arising from the disease itself, specific and supportive treatments adverse effects, as well as underlying patient complexities. The preoperative period demands a comprehensive, systematic assessment that identifies and optimises disease- and treatment-related complications, quantifies frailty, addresses anaemia through patient blood management principles, and stratifies thrombotic risk. Intraoperative management requires careful individualisation of anaesthetic technique in the absence of multiple myeloma-specific evidence-based guidelines, with particular vigilance directed at airway complexity, haemodynamic instability, heightened bleeding risk, and challenging pain management. Postoperative care must sustain these principles, with early attention to renal function, pain control, thromboprophylaxis, and the prompt recognition of the infectious complications to which this population is disproportionately vulnerable. Unfortunately, the literature on anaesthesia in the context of multiple myeloma is sparse, largely limited to isolated case reports. As a consequence, this article's predominantly PubMed-based search strategy (included in supplementary materials) required a wide search to allow for perioperative recommendations that are necessarily extrapolated from broader oncological, haematological, and surgical evidence. As such, this article offers a comprehensive review of anaesthetic considerations in multiple myeloma, addressing an important gap in current practice guidance.

## Graphical abstract

**Figure:**
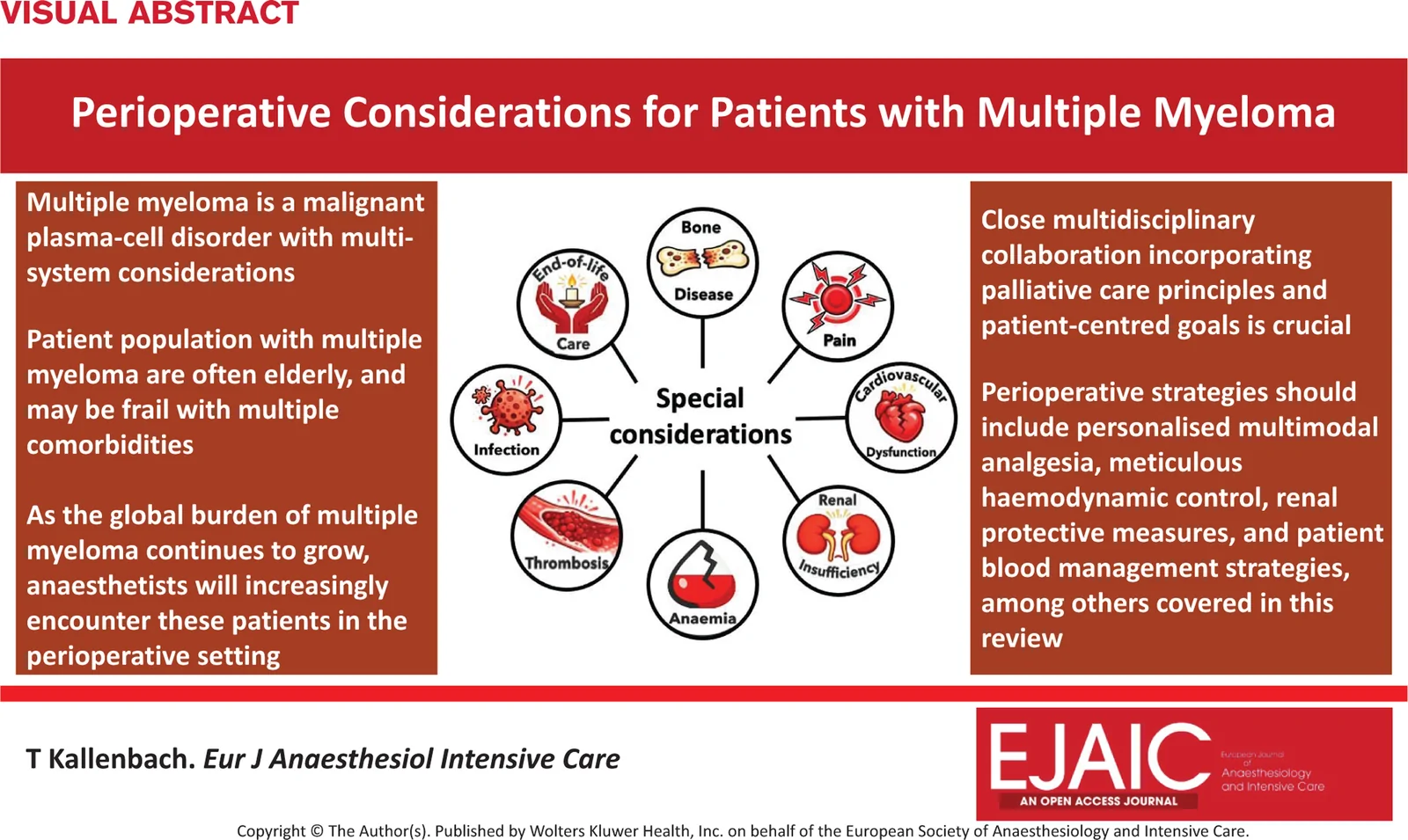

**KEY POINTS**

- The anaesthetist may increasingly encounter a patient with multiple myeloma requiring surgery for disease-related complications, particularly pathological fractures, or incidentally for unrelated procedures.
- The patient with multiple myeloma may be on antimyeloma therapies including chemotherapy and immunotherapy, which have their own considerations.
- In addition to osteolytic bone disease, patients may present with multisystem complications such as renal insufficiency, anaemia, immunocompromise, thrombotic events, and cardiovascular dysfunction.
- Perioperative challenges include complex pain management, preventing exacerbation of renal impairment, minimising infection risk, and mitigating both thrombotic and bleeding risks.

## Perioperative considerations for patients with multiple myeloma

Currently our earliest official record of multiple myeloma (MM) dates back to 1844, documenting a 39-year-old woman who suffered from lethargy and several fractures.^1^ Her condition was recorded as one in which ‘earthy matter of the bone is absorbed and thrown out by the kidneys in the urine’, a remarkably intuitive characterisation of the complex malignancy we recognise today.^1^

Since then, MM incidence has risen markedly worldwide, mainly attributed to population growth and ageing.^2^ Incident cases of MM are projected to increase by at least 71% by 2045.^3^ In addition to delayed diagnosis, reduced access to newer therapies and expensive drugs in lower socio-economic regions adversely affects outcomes for the affected patients.^2^^–^^7^ In 2022, there were approximately 121 000 deaths due to MM, which is projected to rise to 218 000 in 2045, representing an estimated 79% increase in mortality.^3^ Males are more likely to be afflicted than females.^8^^,^^9^ The affected age group is predominantly the elderly; however certain factors, such as HIV, are associated with a younger age at onset of the cancer.^10^^–^^14^

MM is due to expansion of malignant plasma cells predominantly in the bone marrow, with excess production of a monoclonal immunoglobulin or free light chains that may be detected in the serum or urine.^11^ MM usually progresses from monoclonal gammopathy of undetermined significance (MGUS), which is an asymptomatic premalignant state.^11^^,^^15^ There is an intermediate state that may occur before MM called smoldering multiple myeloma.^15^^,^^16^ MM diagnostic criteria, as defined by the International Myeloma Working Group, specify the presence of either greater than 10% abnormal clonal cells in the bone marrow or biopsy-confirmed presence of plasmacytoma, combined with at least one myeloma-defining event from either the CRAB or SLiM criteria, as detailed in Table 1.^15^^,^^17^ Unfortunately, the diagnosis of MM is often delayed as the symptoms tend to be nonspecific, which leads to early morbidity and mortality.^8^^,^^18^

**Table 1:** Myeloma defining features^15^^–^^17^

CRAB criteria	**C**alcium elevation (hypercalcaemia) **R**enal insufficiency **A**naemia **B**one lesions	Calcium >2.75 mmol l^−1^ or >0.25 mmol l^−1^ above the upper limit of normal Creatinine clearance <40 ml min^−1^ or serum creatinine >177 μmol l^−1^ Hb <10 g dl^−1^ or >2 g dl^−1^ below lower limit of normal At least one osteolytic lesion picked up by radiography, CT or PET
SLIM criteria	**S**ixty percent **Li**ght chain **M**RI	≥60% clonal bone marrow cells Ratio of involved to uninvolved serum free light chain ≥100 More than one focal lesion (>5 mm each) seen on MRI

## Pathogenesis

**Fig. 1:**
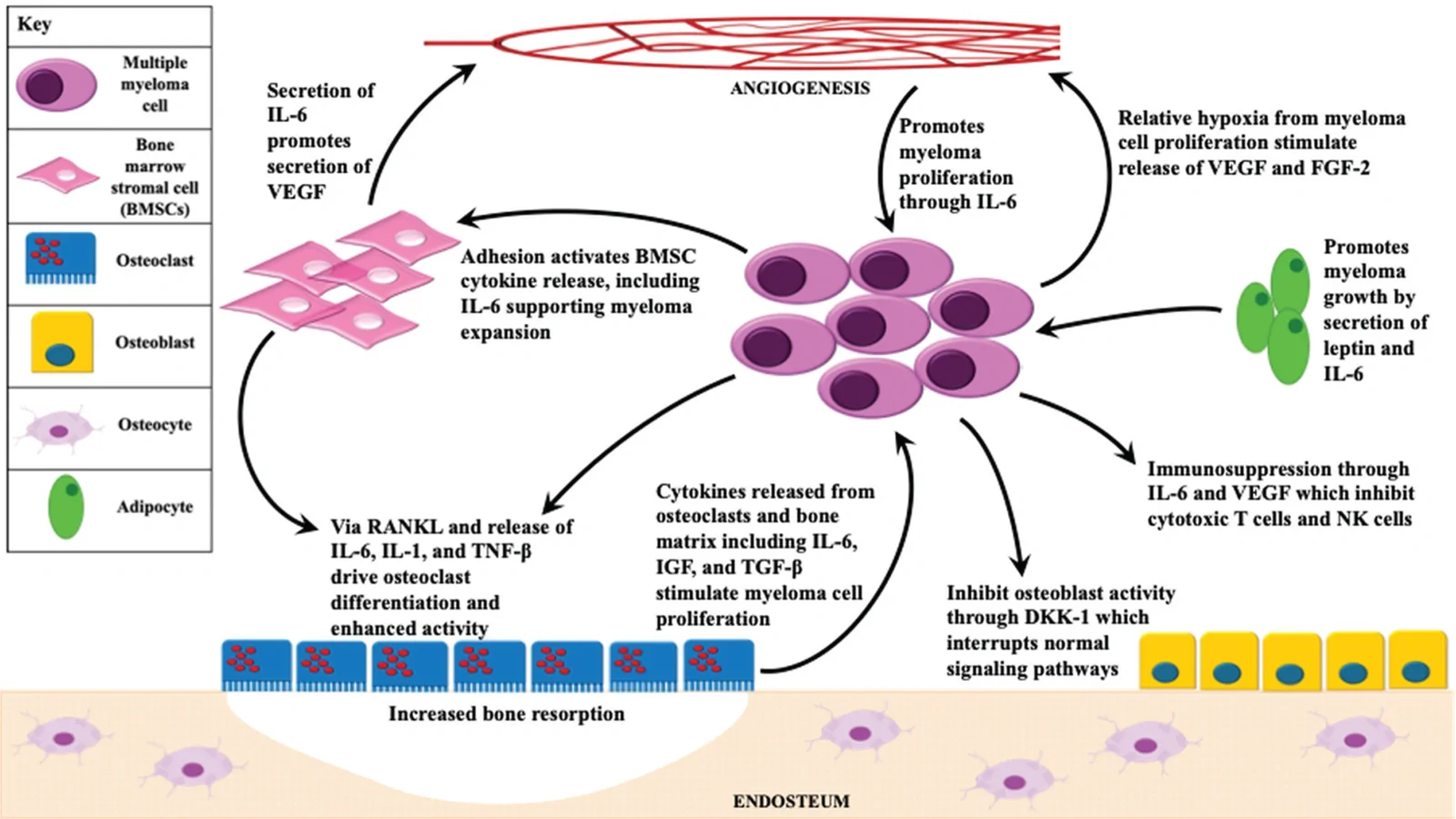
Simplified schematic of the pathogenesis of multiple myeloma.^10,17,19^

BMSCs, bone marrow stromal cells; DKK-1, dickkopf-1; FGF-2, fibroblast growth factor-2; IGF, insulin-like growth factor; IL-1, interleukin 1; IL-1, interleukin 1; IL-6, interleukin 6; NK cells, natural killer cells; THrP, parathyroid hormone-related peptide; RANKL, receptor activator of nuclear factor kappa-β ligand; TGF-β, transforming growth factor beta; TNF-β, tumour necrosis factor beta; VEGF, vascular endothelial growth factor.

While the aetiology of MM is still unclear, there are clear contributing factors that have been identified in its pathogenesis.^19^ Rather than stemming from a single genetic mutation, MM results from a series of genetic abnormalities, with clonal evolution over time decided by the bone microenvironment and selection pressure from the body defences and treatments initiated.^10^^,^^11^^,^^17^^,^^19^ The abnormal plasma cells of MM stimulate the bone marrow stromal cells to release cytokines that promote osteoclast proliferation and growth.^19^ Myeloma cells release mediators that both enhance osteoclast activity and suppress osteoblast function.^10^^,^^19^ Osteoclasts thereby produce unopposed bone destruction, while also releasing cytokines that promote myeloma plasma cell growth, which perpetuates a vicious self-reinforcing feedback cycle.^10^^,^^17^^,^^19^ Bone marrow tissue hypoxia promotes angiogenesis through cytokines such as vascular endothelial growth factor released by the rapidly proliferating plasma cells and stromal cells.^11^^,^^19^ Angiogenesis in turn creates another self-reinforcing feedback loop that supports further growth of these cell groups.^19^^,^^20^ Myeloma cells exert direct immunosuppressive effects and demonstrate resistance to apoptosis.^10^^,^^17^ The bone marrow microenvironment is key in the MM pathogenesis, with various cells such as stromal cells, lymphocytes, and adipocytes releasing factors that support myeloma cell proliferation and disease progression.^11^^,^^17^^,^^19^^,^^20^ This process is illustrated by Fig. 1, although the pathogenesis of MM is substantially more complex than can be captured by this simplified overview.

## Treatment

Currently this disease is incurable, therefore management strategies primarily focus on prolonging survival while optimising patients’ quality of life.^8^^,^^10^ Even with treatment, relapses are common as the cancer progresses.^4^^,^^10^

The mainstay of disease-specific treatment comprises of chemotherapeutic and immunomodulating drugs in combination with autologous stem cell transplantation (ASCT). ASCT itself has been shown to be critical in improving event-free and overall survival.^5^^,^^10^ Unfortunately in some regions widespread access to ASCT is limited, with reduced enrolment in drug trials or funding for newer chemotherapeutic agents in lower socioeconomic environments.^4^^–^^7^^,^^21^

Treatment for a newly diagnosed patient typically begins with induction therapy administered over 3–4 cycles.^15^ Induction regimens commonly entail a steroid (dexamethasone or prednisolone), a proteasome inhibitor (such as bortezomib) and an immunomodulatory drug (thalidomide or lenalidomide) or an alkylating drug (such as cyclophosphamide).^5^^,^^8^^,^^10^ Newer guidelines recommend a quadruplet regimen, which would include monoclonal antibodies such as daratumumab or isatuximab.^15^^,^^22^ Table 2 lists some of the potential adverse effects of certain first-line therapeutic drugs. Stem-cell mobilisation is typically achieved using G-CSF-based strategies to augment stem-cell yield before ASCT.^4^ Haematopoietic stem cells are harvested from the peripheral blood, cryopreserved, and subsequently reinfused following high-dose chemotherapy, most commonly melphalan. ^4^^,^^10^ Maintenance therapy, usually involving a single agent, aims to consolidate treatment response, delay disease progression, and extend survival.^5^^,^^10^ While the above regimen is described, there exist significant variations in treatment protocols and drug availability between medical centres and geographic regions.

**Table 2:** Adverse effects of commonly used chemotherapeutic agents^10^^,^^25^^–^^30^

Drug	Adverse effects
Dexamethasone, Prednisolone	Hyperglycaemia, psychiatric reactions, hypertension, increased susceptibility to infections, adrenal suppression, osteoporosis, dyslipidaemia, myopathy, gastritis, peptic ulcers, thrombocytosis
Bortezomib	Peripheral neuropathy, myelosuppression (anaemia, thrombocytopaenia, neutropenia), hyperglycaemia, hypophosphataemia, anxiety, blurred vision, tinnitus, tachycardia, hypotension, hypertension, renal dysfunction, myalgia, tumour lysis syndrome
Thalidomide	Teratogenicity, neutropenia, venous thromboembolism, orthostatic hypotension, left ventricular systolic impairment, peripheral neuropathy, somnolence, Stevens-Johnson syndrome, reactivation of latent viral infections
Lenalidomide	Teratogenicity, myelosuppression, venous thromboembolism, rash, increased risk of secondary malignancy, peripheral neuropathy, somnolence
Cyclophosphamide	Haemorrhagic cystitis, SIADH, cardiotoxicity, myelosuppression
Daratumumab, Isatuximab	Infusion-related reactions, interference with blood product compatibility testing, increased risk of infections, potential cardiac and pulmonary toxicity including venous thromboembolism, atrial arrhythmias, hypertension
Etoposide	Bronchospasm, worsening of cardiac function in those with underlying cardiac disease, myelosuppression, anaphylaxis, increased risk of secondary malignancy, gastrointestinal upset
G-CSF (filgrastim)	Bone pain, musculoskeletal pain, gastrointestinal upset, fatigue, headache, anaemia, thrombocytopenia, hypersensitivity reactions, ARDS, haemoptysis, splenomegaly and splenic rupture
Melphalan	Myelosuppression, increased risk of infection, mucositis, increased risk of secondary malignancy

ARDS, acute respiratory distress syndrome; G-CSF, granulocyte colony-stimulating factor; SIADH, syndrome of inappropriate antidiuretic hormone secretion.

Some patients may unfortunately not be eligible for ASCT, with factors such as age, frailty, and presence of comorbidities acting as exclusionary criteria, and would likely receive a limited chemotherapeutic regimen as tolerated.^4^^,^^5^^,^^10^ Treatment regimens for relapsed/refractory MM are more varied, depending on patient factors, disease features, and drug availability.^10^^,^^15^^,^^22^ Promising novel therapies for relapsed/refractory MM include chimeric antigen receptor T-cell (CAR T-cell) therapy and bispecific antibodies, which are further detailed in Table 3.^10^^,^^23^ Alongside disease-specific interventions, comprehensive supportive care remains an essential component of patient comfort and disease management.^5^^,^^10^^,^^24^

**Table 3:** T-cell-engaging immunotherapies^31^^–^^35^

	Mechanism of action	Notable adverse effects
Bispecific antibodies (BsAbs)	Engineered fusion proteins designed to bind two targets simultaneously: tumour-associated surface antigen and patient T cell CD3 antigen Cytolytic immune synapse formed activates the T cell and triggers degranulation, culminating in direct tumour cell lysis	Cytokine release syndrome (CRS) - Symptoms: fever, rigor, malaise, fatigue chills, headache; progression to hypotension, hypoxia, tachycardia, multiorgan dysfunction in severe cases - Management depending on severity: analgesics, antipyretics, fluid replacement, IL-6 inhibitor therapy, dexamethasone, organ support, ICU admission if needed - Worsening of capillary leak and vasodilation with anaesthesia - Use of vasopressors encouraged, avoid prolonged starvation, avoid hypoxaemia and hypercarbia
Chimeric antigen receptor modified T-cell therapy (CAR T-cell therapy)	Genetic modification of patient's own T cells to express a chimeric antigen receptor, which is an engineered surface protein designed to recognise and bind a specific tumour-associated antigen Once the CAR engages its target, patient T-cell is activated, killing the tumour cell Note: this treatment requires leukapheresis, through which autologous T cells are harvested, genetically modified and then reinfused	Neurotoxicity including immune effector cell-associated neurotoxicity syndrome (ICANS) - Symptoms: confusion, dysphasia, impaired fine motor skills, cognitive slowing; progression to drowsiness, headache, seizures and cerebral oedema in severe cases - Neurotoxicity can occur with cranial nerve palsy, peripheral neuropathy, or Parkinsonian-like movement disorders - Management: haloperidol or lorazepam if agitated, dexamethasone, antiepileptics, with ICU care and CSF drainage in severe cases - Approach as high risk for raised intracranial pressure including avoiding hypoxia, hypercarbia, hyperthermia, with head elevation where possible; continuation of anticonvulsants perioperatively Infections - Symptoms: fever, tachycardia, infection-specific - Bacterial, viral, or fungal, with respiratory system commonly affected - Management: prophylaxis is essential at immunotherapy initiation, low index of suspicion and early initiation of treatment - Compounded by cytopaenias and hypogammaglobulinaemia which are also known adverse effects of T-cell-engaging immunotherapies - Strict asepsis anaesthesia techniques perioperatively

## Specific considerations

### Bone disease

Osteolytic bone disease is one of the defining characteristics of MM, often resulting in bone pain and may present as pathological fractures.^10^^,^^24^^,^^36^ Management of MM bone disease can include antiresorptive therapy (typically bisphosphonates or denosumab), radiotherapy, interventional procedures, and surgery.^24^^,^^37^

Antiresorptive therapy aims to prevent further bone loss and reduces the risk of life-threatening hypercalcaemia, improves bone pain, and decreases the incidence of pathological fractures.^11^^,^^24^^,^^26^ There are concerning side-effects of bisphosphonate and denosumab use including medication-related osteonecrosis of the jaw (MRONJ), as well as renal injury from bisphosphonate use.^19^^,^^26^^,^^36^ MRONJ is rare, but should it occur the patient will present with nonhealing necrosis of the mandible or maxilla exhibited by pain, swelling and potentially infection, all of which would be an airway concern for the anaesthetist.^38^^,^^39^

### Pain

Pain is one of the most prevalent and debilitating symptoms for patients with MM, yet often poorly managed.^40^^,^^41^ Due to the variable nature of pain, regular assessment of pain both preoperatively and postoperatively is recommended using a validated patient-reported rating scale.^37^^,^^42^ Effective and personalised analgesic strategies are crucial as poorly managed postoperative pain contributes to increased morbidity, poorer recovery, and decreased quality of life.^41^^–^^43^

Perioperative pharmacological pain management strategies for MM tend to follow the WHO analgesic ladder with progressive steps from nonopioid analgesics to weak opioids to strong opioids.^37^^,^^44^ Pain specialists and psychologists can provide valuable interventions, advice, and support to optimise pain management perioperatively. ^8^^,^^40^ For spinal column involvement, patients can undergo low-dose radiation which provides marked pain relief with a relatively small incidence of side effects.^11^^,^^19^^,^^36^ In appropriate cases, minimally invasive procedures such as vertebroplasty and kyphoplasty contribute favourably to symptomatic improvement.^5^^,^^10^^,^^36^ Peripheral neuropathy may necessitate adjustments of certain neurotoxic antimyeloma agents, in addition to supportive measures and analgesics.^45^^,^^46^

### Cardiovascular dysfunction

When analysing deaths directly related to MM itself, cardiovascular causes constitute a major contributor to overall mortality among affected patients.^47^^,^^48^ Manifestations of cardiovascular dysfunction include hypertension, accelerated ischaemic heart disease, and conduction disorders.^29^^,^^49^ Approximately 30% of patients with MM have amyloid light chain deposits.^29^^,^^50^^,^^51^ These over-produced light chains by MM plasma cells misfold and collect in cardiac, as well as renal, gastrointestinal, pulmonary, neural, or other tissues, causing damage from structural distortion as well as direct cellular toxicity.^29^^,^^49^ Cardiac, as well as renal, manifestations occur frequently with amyloidosis, with cardiovascular damage often presenting as diastolic dysfunction, heart failure with preserved ejection fraction, arrhythmias particularly atrial fibrillation, intracardiac thrombi, or autonomic dysfunction.^29^^,^^49^^,^^52^

### Renal insufficiency

Renal dysfunction is frequently seen in MM, and is associated with higher disease burden and more aggressive disease progression.^10^^,^^23^^,^^53^ Perioperatively, application of renal-protective strategies is essential, even in patients with apparently normal baseline function, as underlying vulnerability persists.^54^ Recommended strategies include avoiding nephrotoxic drugs as well as meticulous maintenance of hydration and normocalcaemia.^5^^,^^11^^,^^54^ Hypercalcaemia exacerbates renal injury and should acute severe hypercalcaemia occur perioperatively, this medical emergency requires prompt management with isotonic fluid resuscitation, steroids and bisphosphonates.^5^^,^^11^^,^^19^ In the presence of myeloma-related acute renal injury, antimyeloma treatment and fluid therapy are commenced urgently, with plasmapheresis or dialysis instituted if required as a bridging therapy.^23^ Other indications for dialysis include severe volume overload, refractory acid-base disturbances, and persistent electrolyte disorders as with any other underlying cause of acute kidney injury.^23^^,^^55^ Perioperative medication adjustment for renally metabolised or excreted drugs should be done when renal dysfunction is present.^23^

### Anaemia

Anaemia, predominantly normochromic normocytic subtype, affects approximately two-thirds of patients with MM, leading to a characteristic MM symptom of fatigue.^19^^,^^56^^,^^57^ Anaemia should be managed using patient blood management principles of haemoglobin optimisation, reducing blood loss through surgical and anaesthesia techniques, and improving patient tolerance of anaemia.^58^^–^^60^

### Thrombosis

Patients with MM have a substantially elevated risk of thrombosis compared with the general population, making preoperative individualised risk assessment advisable.^10^^,^^56^ Depending on the level of risk, patients may be considered for prophylactic aspirin alone, or in combination with low molecular weight heparin, fondaparinux, or warfarin.^10^^,^^11^ Thromboprophylaxis is recommended for six months following initiation of chemotherapy.^11^^,^^56^ Hyperviscosity syndrome, a rare complication classically composed of visual disturbances, mucosal bleeding and neurological deficits, increases thrombotic but also paradoxically bleeding risk.^61^^,^^62^ Direct oral anticoagulants, such as rivaroxaban, have become an alternative for outpatient thromboprophylaxis, which will need perioperative management according to standardised guidelines.^24^^,^^63^^,^^64^

### Infection

Patients with MM are highly susceptible to infections, especially pneumonia and septicaemia, which require early diagnosis and prompt treatment as it is otherwise related to significant morbidity and mortality.^24^^,^^65^^,^^66^ It is important to note the increased risk of bloodstream infections related to central venous catheters including chemotherapy ports, dialysis catheters, and peripherally inserted central catheters (PICC), underscoring the importance of meticulous line care on the part of the anaesthetist.^67^^,^^68^ Individuals who are at risk for developing severe infections are typically those with poor functional status, comorbidities, and renal dysfunction, with high tumour load and increased lactate dehydrogenase serving as useful markers.^38^

While early recognition and prompt intervention is important, preventive strategies represent a cornerstone of care. There are variations in protocols, but prior to surgery the patient with MM may already be on antibacterial prophylaxis, such as levofloxacin or trimethoprim-sulfamethoxazole, and antiviral prophylaxis, such as acyclovir or valaciclovir.^10^^,^^11^^,^^24^^,^^38^ Elective surgery should be deferred in the presence of neutropenia until adequate haematological recovery has been achieved.^30^

### End-of-life care

Early discussions regarding prognosis and goals of care are essential for patients with MM, with clear documentation of end-of-life preferences including advance medical directives.^8^^,^^69^ As MM is associated with poor quality of life in both the initial as well as advanced stages of the disease, often accompanied by psychological distress manifesting as anxiety and depression, ongoing proactive engagement with optimising patient-reported outcomes is crucial.^70^^,^^71^ Due to the terminal nature of MM, procedures carried out are often supportive and palliative in nature, particularly once disease relapse has occurred.^72^ Effective end-of-life care requires close collaboration with palliative care teams, alongside active involvement and support of family members and caregivers.^70^^,^^73^

## Perioperative management

As the above specific considerations have shown, perioperative management of MM requires careful consideration of multiple factors arising from both the disease itself and its treatment. Optimal perioperative care is best delivered through a multidisciplinary approach, involving not only the surgical and anaesthetic teams but also the treating haematologist-oncologist, together with allied health professionals such as psychologists, physiotherapists, occupational therapists, dieticians, social workers, and palliative care team.^8^^,^^73^

### Preoperative

Patients with MM may present for surgery either for specific MM-related complications, such as pathological fractures, or where the cancer is incidental to the primary surgical condition. Unfortunately, emergency presentation represents one of the commonest routes to MM diagnosis, and is associated with more advanced disease and poorer survival.^74^^,^^75^ Acute spinal cord collapse represents a surgical emergency, while typical elective orthopaedic procedures include spinal decompression, reconstruction, or internal fixation, and long bone surgery, particularly intramedullary nailing, plating, or cementation of the femur or humerus.^72^^,^^76^^–^^78^ Surgical intervention for the prevention or fixation of pathological fractures has been shown to improve pain, quality of life, and overall survival.^11^^,^^19^^,^^36^ However, perioperative risk is increased owing to factors such as frailty, anaemia, renal dysfunction, and other disease- and treatment-related complications.^19^^,^^36^^,^^72^ Worryingly, haematogenous seeding of MM has been linked to surgical procedures.^79^^,^^80^

A meticulous preoperative assessment is therefore essential. MM patients frequently present with significant comorbidities and sequelae such as hypertension, diabetes, congestive cardiac failure, ischaemic heart disease, chronic lung or renal disease, with advancing age compounding this burden.^81^^,^^82^ In keeping with the predominant patient demographic of MM, preoperative frailty assessment using a validated scoring system is advised.^83^ The spine represents the most common site of MM bone metastasis, and while neurological deficits rarely occur, a focused neurological assessment is warranted. ^72^^,^^75^^,^^76^ Polypharmacy is common, and where renal dysfunction is present, furosemide, ACE inhibitors, and angiotensin-receptor blockers necessitate careful review with consideration given to withholding or substituting these agents preoperatively.^23^

Preoperative blood investigations should include full blood count, electrolytes including calcium, urea, creatinine, and coagulation studies.^22^^,^^55^^,^^84^^–^^86^ ProBNP (brain natriuretic peptide) is an important screening tool where cardiac dysfunction is a concern, and in addition to troponin should be routinely done if amyloidosis is suspected.^29^^,^^85^^,^^87^ Concerning comorbidities or new-onset cardiovascular symptoms warrant further investigations such as ECG, echocardiography, or cardiac MRI as appropriate.^29^^,^^49^^,^^53^

Osteolytic lesions typically occur in the axial skeleton (skull, spine, pelvis), the long bones (humerus, femur), as well as in the ribs and sternum.^19^^,^^72^ If x-rays are done, these lesions will appear as hyperlucent “punched-out” defects accompanied by cortical thinning, and when multiple lesions of the calvarium are present, they produce the classic “pepper pot” appearance of the skull.^19^^,^^88^

Currently, whole body low-dose CT, MRI or PET-CT is recommended as first-line imaging for skeletal lesions.^72^^,^^85^^,^^88^ These investigations may additionally identify extramedullary disease, arising from myeloma subclones that have escaped the bone marrow microenvironment.^80^^,^^88^^,^^89^ Although rare at diagnosis, extramedullary MM increases in frequency with successive relapses, carrying a poorer prognosis, and may be found deposited in the skin, pleura, lymph nodes, liver, kidneys or central nervous system. ^80^^,^^89^^,^^90^ Given the predisposition to renal impairment in this population, the risk of contrast-induced nephropathy should be carefully considered when planning the above investigations.^23^

Significant comorbidities should be optimised before surgery, with fasting periods minimised and adequate hydration maintained to preserve renal function.^55^^,^^85^^,^^91^ Preoperative pain control should follow a stepwise approach, commencing with paracetamol and weak opioids, escalating to stronger opioids such as morphine, buprenorphine, and fentanyl for refractory pain, with nonsteroidal anti-inflammatory drugs (NSAIDs) avoided given their nephrotoxic potential. ^40^^,^^53^^,^^56^ For chronic, persistent, or neuropathic pain, additional agents such as pregabalin, duloxetine, or amitriptyline should be considered.^37^^,^^53^^,^^92^

Any reversible causes of anaemia should be investigated and appropriately managed, such as iron supplementation for iron deficiency.^5^^,^^56^ Iron infusions are generally recommended over oral iron therapy as it bypasses the effects of hepcidin as well as the gastrointestinal intolerance associated with chemotherapy.^59^^,^^93^ For symptomatic anaemia with haemoglobin below 10 g dl^−1^, erythropoiesis-stimulating agents have demonstrated efficacy in raising haemoglobin and reducing transfusion requirements, though their use must be balanced against recognised risks, particularly thrombotic complications.^5^^,^^56^^,^^94^ Preoperative pharmacological venous thromboembolism prophylaxis is recommended for patients undergoing major surgery, unless a high bleeding risk contraindicates its use.^63^ Omission of anticoagulation at the appropriate time and avoidance of NSAIDs are essential preoperative measures to mitigate perioperative bleeding risk.^58^^,^^64^

### Intraoperative

In the absence of evidence-based guidelines advocating one anaesthetic technique over another for MM patients, technique selection is guided by individual patient and surgical factors, with case reports documenting successful use of general, neuraxial, and regional anaesthesia.^54^^,^^95^^–^^99^ Regional and neuraxial techniques require caution in the presence of neurological symptoms, and neuraxial anaesthesia may be contraindicated in the presence of severe vertebral destruction or disease-related or treatment-induced coagulopathy.^95^^,^^96^^,^^99^

Airway management may be complicated by axial instability secondary to osteolysis, amyloid-related macroglossia and xerostomia, or MRONJ.^52^^,^^87^^,^^96^^,^^99^ Where cervical involvement is suspected or confirmed, principles applicable to cervical spine injury should be observed, including minimisation of cervical movement and the use of video-laryngoscopy.^100^ Although less frequently affected than the remainder of the axial skeleton, cervical MM carries disproportionately higher morbidity and instability risk, and is typically managed conservatively.^77^ Macroglossia from amyloidosis can result in significant upper airway obstruction requiring CPAP, while less common oesophageal and gastric amyloid involvement, manifesting as reflux, dysphagia, vomiting, and distension, create concerns for aspiration.^52^^,^^101^

Intraoperative pain management remains a particular challenge, compounded by factors such as preexisting bone pain, NSAIDs contraindications, renal dysfunction affecting drug metabolism, and limitations to neuraxial techniques.^102^ Prolonged opioid exposure from chronic pain may result in opioid tolerance or opioid-induced hyperalgesia, rendering dose escalation potentially ineffective, while increasing the risk of chronic postsurgical pain.^103^ Multimodal analgesia incorporating at least two nonopioid strategies is recommended, with adjuvant agents including ketamine, lignocaine infusion, dexmedetomidine, paracetamol, and magnesium, alongside regional nerve blocks where appropriate, offering valuable opioid-sparing options. ^44^^,^^102^^,^^103^ Low-dose ketamine has demonstrated particular benefit in opioid tolerance and hyperalgesia, while lignocaine and dexmedetomidine confer additional anti-inflammatory properties. ^44^^,^^102^^–^^104^ Magnesium correction should be considered where hypomagnesaemia is suspected, given its potential association with pain alleviation in cancer patients.^44^^,^^105^

In complex cases, invasive arterial pressure monitoring or advanced cardiac output monitoring should be considered to guide haemodynamic management and volume assessment.^55^ Where autonomic dysfunction is present, whether secondary to amyloidosis or treatment agents, intraoperative cardiovascular stability may be challenging to maintain, particularly in patients receiving beta-blockers, ACE inhibitors, angiotensin-receptor blockers, or vasodilators preoperatively.^45^^,^^49^^,^^52^^,^^87^ Renal protection strategies, such as those detailed in KDIGO guidelines, include a target glucose range of 7.8–10 mmol l^−1^ to avoid hyperglycaemia, avoiding intraoperative nephrotoxic drugs such as aminoglycoside antibiotics, maintaining euvolaemia, and preventing hypotension from anaesthesia or surgical haemodynamic fluctuations.^55^^,^^91^

Blood loss represents a significant intraoperative concern, with increased transfusion requirements well documented in MM orthopaedic surgery, and bleeding risk in spinal surgery demonstrated to exceed that of other malignancies.^99^^,^^106^^–^^108^ Contributing mechanisms include MM-mediated thrombocytopaenia, acquired von Willebrand syndrome, DOAC use, and antimyeloma therapy side effects, with acquired coagulation factor deficiencies and dysfibrinogenaemia occurring less commonly.^62^^,^^86^^,^^109^

Blood conservation strategies encompass minimally invasive surgical approaches, meticulous haemostasis, careful haemodynamic and fluid management, and avoidance of hypothermia and acidosis.^58^^,^^72^ Tranexamic acid use in metastatic spinal tumour surgery has not been associated with significantly increased thromboembolic events, a valid concern for MM patients, and is suggested where significant blood loss is anticipated.^59^^,^^110^ The use of mechanical compression devices is encouraged to reduce intraoperative thrombotic risk.^84^ Intraoperative cell salvage is a viable option for reducing allogeneic transfusion requirements, provided leucocyte depletion filters are utilised and the associated risks and benefits are discussed with the patient preoperatively.^59^^,^^111^

Intraoperative blood transfusions do need to be considered in patients who have not responded to other therapeutic modalities, those who are acutely symptomatic, or those needing an urgent correction of their anaemia.^5^^,^^56^^,^^112^ The anaesthetist should inform the blood bank if the patient has been receiving daratumumab due to its interference with blood group compatibility testing.^113^ There is some uncertainty on the definitive transfusion threshold in oncology patients. While certain guidelines give a conditional, low-certainty recommendation for a haemoglobin threshold of 7 g dl^−1^ in stable hospitalised patients, others explicitly caution against a threshold-driven approach, advocating instead for clinically guided decision-making that incorporates severity of comorbidities, rate of haemoglobin decline, and the presence of anaemia-related symptoms.^59^^,^^112^^,^^114^^,^^115^ Use of viscoelastic assays such as TEG or ROTEM are encouraged to guide blood product administration, and leucodepleted products should be used due to MM disease- and treatment-related immunosuppression.^84^^,^^112^

Additional intraoperative considerations include meticulous patient positioning to minimise the risk of pathological fractures.^99^ Normothermia should be actively maintained to reduce the associated risks of coagulopathy, altered pharmacokinetics, and postoperative infection.^84^^,^^99^^,^^116^^,^^117^ To further mitigate infection risk, strict aseptic technique must be maintained throughout and prophylactic antibiotics should be administered timeously.^95^^,^^96^^,^^99^

### Postoperative

The frequently reported postoperative complications include infectious events, particularly pneumonia, urinary tract infections, and surgical site infections, as well as thromboembolic complications and acute renal failure.^78^^,^^106^^,^^107^^,^^118^ Many of the principles outlined above should be continued into the postoperative period. Pain management is often an extension of the principles used preoperatively and intraoperatively, with methadone and buprenorphine use for refractory pain.^40^^,^^104^

Elevated creatinine levels are associated with a significantly increased risk of postoperative complications.^106^ Given the inherent vulnerability of the kidneys in MM, meticulous attention to renal protection is essential including maintenance of normoglycaemia, and avoidance of nephrotoxic drugs and radiocontrast agents.^55^^,^^91^^,^^119^ The prevention of hypotension may warrant the use of advanced haemodynamic monitoring postoperatively in clinically complex cases to guide volume optimisation and maintain adequate renal perfusion pressure.^119^

Postoperative haemoglobin should be monitored, with serial assessment continued for at least three days following major surgery or significant intraoperative blood loss.^120^ Beyond the patient blood management strategies outlined above, physiological tolerance to anaemia, if present, may be augmented by maintenance of adequate haemodynamic status and organ perfusion, supplemental oxygen therapy, and judicious bed rest as clinically indicated.^60^^,^^121^ The anaesthetist should vigilantly monitor for ongoing blood loss, such as through surgical drains and repeated blood sampling.^60^^,^^120^

Early postoperative re-initiation of anticoagulant therapy is recommended, with extended prophylaxis indicated after high-risk procedures without significant bleeding complications, or in patients independently assessed to be at high thromboembolic risk.^84^ Mechanical prophylaxis, such as intermittent pneumatic compression or graduated compression stockings, should be employed in combination with pharmacological agents where feasible, or as the sole prophylactic modality in the setting of active bleeding until haemostasis is achieved.^63^^,^^84^

## Conclusion

MM presents the anaesthetist with a considerable perioperative challenge, with its broad spectrum of disease manifestations and treatment-related complications. A thorough understanding of MM-specific perioperative strategies, including effective pain management, renal protection, patient blood management, and the prevention of thrombotic and infectious complications, is therefore essential. As advances in antimyeloma therapy continue to extend patient survival, the anaesthetist must be well equipped to deliver high-quality, individualised perioperative care to this growing and increasingly complex patient population.

## Supplementary Material

Supplemental Digital Content
