# Supplementary material for: Perioperative considerations for patients with multiple myeloma
Source: Eur J Anaesthesiol Intensive Care. 2026 Jul 8;5(4):e0124. doi: 10.1097/EA9.0000000000000124 (PMC13568272; doi:10.1097/EA9.0000000000000124)
Supplement: Supplementary file 1 [file ejaic-5-e0124-s001.docx]

**Search strategy**

Predominantly PubMed-based search strategy, supplemented with Web of Science and Google platforms.

No date restrictions, although more recent articles were favoured, and time limit of 10 years was occasionally placed to find these articles

Language restriction to English

The below search terms were used in varying combinations:

myeloma; multiple myeloma; cancer; oncology; oncological; haemotological; hematological; haematology; hematology; perioperative; preoperative; pre-operative; intraoperative; intra-operative; postoperative; anaesthesia; anesthesia; surgery; surgical; guideline; guidelines; protocol; management; treatment; investigation; prophylaxis; diagnosis; incidence; prevalence; human immunodeficiency virus; demographics; socio-economic; low-income; pathogenesis; osteoblast; osteoclast; angiogenesis; treatment; chemotherapy; chemotherapeutics; immunotherapy; autologous stem cell transplantation; ASCT; induction; maintenance; relapse; T-cell; BCMA; bispecific antibodies; BsAbs; chimeric antigen receptor modified T-cell therapy; CAR T-cell therapy; osteolysis; osteolytic bone disease; antiresorptive; bisphosphonates; denosumab; medication-related osteonecrosis of the jaw; MRONJ; bisphosphonate-induced osteonecrosis of the jaw; BIONJ; pain; analgesia; pain management; acute pain; chronic pain; WHO analgesic ladder; radiation; vertebroplasty; kyphoplasty; cardiovascular; amyloid; amyloidosis; light chain; renal; kidney; nephrotoxic; nephrotoxicity; hypercalcaemia; dialysis; anaemia; anemia; patient blood management; thrombosis; thromboembolism; thromboprophylaxis; hyperviscosity syndrome; direct oral anticoagulants; infections; infectious; central venous catheters; end-of-life care; patient-reported outcomes; palliative care; palliation; multidisciplinary; orthopaedic; spine; femur; humerus; acute spinal cord collapse; haematogenous spread; comorbidities; frailty; frail; renal protection; renal protective; x-ray; extramedullary disease; peripheral neuropathy; neuropathic; iron infusion; hepcidin; blood; transfusion threshold; erythropoietin; erythropoiesis-stimulating agents; general; neuraxial; spinal; regional; regional block; airway; cervical spine; cervical trauma; macroglossia; aspiration; opioid tolerance; opioid-induced hyperalgesia; multimodal; ketamine; magnesium; haemodynamic monitoring; autonomic dysfunction; blood loss; bleeding; tranexamic acid; cell salvage; blood transfusion; daratumumab; antigen; positioning; temperature; hypothermia; complication
